# Phage-Related Ribosomal Proteases (Prps): Discovery, Bioinformatics, and Structural Analysis

**DOI:** 10.3390/antibiotics11081109

**Published:** 2022-08-16

**Authors:** Julia A. Hotinger, Allison Hannah Gallagher, Aaron E. May

**Affiliations:** Department of Medicinal Chemistry, School of Pharmacy, Virginia Commonwealth University, Richmond, VA 23219, USA

**Keywords:** phage-related ribosomal protease, bioinformatics, X-ray crystal structure, antimicrobials, new targets

## Abstract

Many new antimicrobials are analogs of existing drugs, sharing the same targets and mechanisms of action. New antibiotic targets are critically needed to combat the growing threat of antimicrobial-resistant bacteria. Phage-related ribosomal proteases (Prps) are a recently structurally characterized antibiotic target found in pathogens such as *Staphylococcus aureus*, *Clostridioides difficile*, and *Streptococcus pneumoniae.* These bacteria encode an N-terminal extension on their ribosomal protein L27 that is not present in other bacteria. The cleavage of this N-terminal extension from L27 by Prp is necessary to create a functional ribosome. Thus, Prp inhibition may serve as an alternative to direct binding and inhibition of the ribosome. This bioinformatic and structural analysis covers the discovery, function, and structural characteristics of known Prps. This information will be helpful in future endeavors to design selective therapeutics targeting the Prps of important pathogens.

## 1. Introduction

Antibiotic resistance is a continuously worsening global health threat. The prevalence of antibiotic-resistant pathogens continues to increase. The number of novel antibiotics approved by the FDA has decreased over the past 30 years [1]. This imbalance is demonstrated by the CDC’s estimated >2.8 million cases of antibiotic-resistant infections annually in the United States. Out of these infections, over 35,000 result in death [2]. This is a 40% increase in antibiotic-resistant infections compared to 6 years ago [2].

Rather than targeting only pathogenic bacteria, many common antibiotics kill much of the host’s microbiome in addition to the pathogen. Under normal circumstances, these commensal bacteria help keep opportunistic pathogens under control. However, when commensals are depleted, it can increase the risk of secondary infection from opportunistic pathogens. For example, antibiotics patients are at increased risk for *Clostridioides difficile* infections, a bacterium that can cause deadly diarrhea if growth is not controlled [2]. *C. difficile* secondary infections caused 223,900 hospitalizations throughout 2017 in the USA, including 12,800 deaths [2]. These staggering numbers have caused the CDC to classify *C. difficile* as an urgent threat. Other species considered serious threats by the CDC include *Staphylococcus aureus* and *Streptococcus pneumoniae*, with 900,000 and 323,000 cases in the USA annually [2]. Between the number of species that have developed antibiotic resistance and the risk of secondary infections, antibiotic resistance poses a major threat to human health. New mechanisms and targets for treating these infections must be developed that adopt more selective approaches to targeting pathogenic bacteria.

The ribosome is an essential organelle involved in protein translation and has served as one of the most commercially successful antibiotic targets in history [3]. Several classes of antibiotics utilize different mechanisms to target the bacterial ribosome, including tetracyclines [4], oxazolidinones [5,6], macrolides [7,8], and aminoglycosides [9,10]. The goal of these antibiotics is to interfere with the synthesis of proteins essential for the bacteria’s survival. Given the size and complexity of the ribosome, there are likely many additional undiscovered ways to target it, and it remains a fruitful antibiotic target.

An essential component of the ribosome in bacteria such as *S. aureus*, *C. difficile*, and *S. pneumoniae* is ribosomal protein L27. Some species of bacteria, such as these Firmicute pathogens, have a slightly different L27 compared to other species, such as the Proteobacteria *Escherichia coli* [11]. The L27 present in Firmicutes, Tenericutes, and Fusobacteria (long L27) have an N-terminal extension that must be cleaved during ribosomal assembly to produce a functional ribosome [12]. The enzyme that performs this cleavage was discovered to be a part of a new class of cysteine proteases named phage-related ribosomal protease (Prp). Since only a specific subset of bacteria contain Prp, targeting this enzyme could spare many commensal bacteria that do not use it. Sparing commensal bacteria should also reduce selective pressure, slow antibiotic resistance rates, and reduce negative health outcomes associated with microbiome depletion [13]. Thus, Prp may represent a promising narrow-spectrum antibiotic target, and the information provided in this article will be helpful in future endeavors to design selective therapeutics targeting the Prps of important pathogens.

### 1.1. Discovery of Prp & Phage Relation

The discovery of Prp’s function first occurred in a study examining capsid assembly in Staphylococcal phage 80α. Gp46 and gp47 are the major capsid and scaffold proteins, respectively, that assist in assembling the capsid of 80α. The successful incorporation of gp46 and gp47 into the capsid is dependent on a posttranslational cleavage. Some phages encode for “prohead” proteases that will conduct cleavage of structural proteins; however, no such protease is found in 80α [14,15]. This suggests that the protease used to cleave the scaffold proteins originated within the Staphylococcal genome. To test this hypothesis, gp46 and gp47 were expressed in both *S. aureus* and *E. coli*. Cleavage of both capsid and scaffold was observed in *S. aureus* but not *E. coli*, suggesting that this hypothetical protease was endogenous and specific to *S. aureus* [16]. The gp46 and gp47 were then compared to the *S. aureus* proteome to identify similar cleavage sequences and the protease responsible for their cleavage within *S. aureus* (Table 1). The N-termini of gp46 and gp47 were highly similar to that of ribosomal protein L27 [16,17].

### 1.2. Ribosomal Protein L27

**Figure 1:**
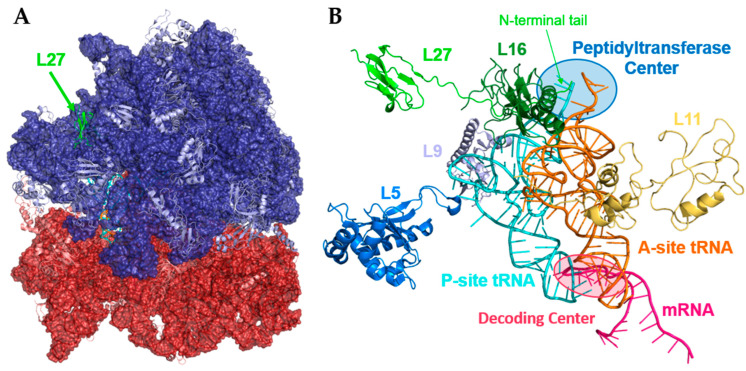
Ribosomal protein L27. (**A**). Ribosomal proteins (RPs) in *E coli*. The large subunit (50S, blue) is made of rRNA (dark blue) and 34 RPs (light blue). The small subunit (30S, red) contains rRNA (red) and about 21 RPs (salmon). L27 (green) is mainly located in the 50S ribosomal subunit (blue) but has an N-terminal tail that reaches the peptidyltransferase center (PTC) at the interface between the 50S and 30S (red) subunits. (**B**). Large subunit-associated RPs directly interacting with tRNA. This includes L27’s N-terminus (green) stabilizing the 3′ ends of tRNA in both the A-site (orange) and P-site (cyan). PDB code: 4V6F. All images produced with PyMOL Molecular Graphics System, Version 2.0 Schrödinger, LLC.

The bacterial ribosome is comprised of ribosomal RNA (rRNA), and ribosomal proteins (RPs) organized into two subunits, the 50S and 30S (Figure 1) [18]. These proteins assist in the structural stability of the ribosome, mRNA translation, and protein assembly [19]. One source of confusion lies in the naming convention of these proteins. For example, the eukaryotic RP L27 is homologous to RP L15 in bacteria, while the RP L27 in bacteria has no known homolog in eukaryotes [20,21]. Of the 54 RPs found in the bacterial ribosome, approximately 15 are always considered essential and 22 are considered non-essential, although deletion of non-essential RPs typically results in significant issues such as truncated protein production, protein aggregation, and severely stunted growth [20]. Of note, the number and composition of RPs considered essential differ slightly in each bacterial species and is often highly dependent on growth conditions, such as temperature and nutrients. For example, L21, L27, L30, S9, S15, and S17 are essential in *B. subtilis* but not *E. coli*, while the opposite is true for L22, L23, L28, and S1 [20].

In the case of RP L27 (Figure 1, green), its N-terminal tail stabilizes the 3′ end of tRNA in the A- and P-site within the peptidyltransferase center (PTC) of the ribosome (Figure 1B), allowing for efficient translation of mRNA [12,22,23]. The only other RP known to interact directly at the PTC is L16, which acts mainly to stabilize A-site tRNA [20]. Non-direct contributing RPs to PTC activity include L1 (exit gate for tRNA), L5 (stabilization of P-site tRNA at elbow region), L9 (P-site tRNA stability), L11 (stringent response A-site tRNA deacetylation sensing), and S12 (decoding codon of A-site tRNA) [20,24]. Some also consider RPs L4, L17, L22, L23, L29, and L32 as indirect actors of the PTC, as they form an exit tunnel to stabilize the peptide as it is produced and guide it out of the ribosomal complex [20,25].

In *S. aureus,* L27 is considered an essential protein as knockouts result in non-viable cells and complementation on a plasmid was able to restore function [16]. The essentiality of L27 in Firmicutes other than *B. subtilis* and *S. aureus* has not been experimentally proven. The direct interaction of L27 with the 3′ end of tRNA indicates an important role in translation, supporting the notion of their essentiality [12,22,23]. Converse to this, Yutin et al. showed that the gene for L27 does not duplicate in a phylogenetic study of over 50,000 RPs, suggesting a lack of need, as proteins with paralogs are more likely to be essential [26]. While fundamentally important, the essentiality of L27 may not be a significant problem in the context of infection treatment. Significant growth defects and increased protein aggregation are seen when L27 is knocked out in bacteria that do not require L27 for viability, such as *E. coli* [27]. These negative effects are also seen when the N-terminus is truncated by three amino acids or when L27 is phosphorylated, emphasizing the importance of L27’s interaction with tRNA for reliable protein translation [28,29]. This stagnation in growth may allow Prp inhibitors to act as nearly bacteriostatic agents for pathogens in which L27 is not essential for growth and facilitate immune system clearance [30].

### 1.3. Prp as a Cleavage Enzyme

Some bacteria, including *S. aureus*, encode “long” L27 with a supernumerary cleavage sequence on the N-terminus that is not seen in others such as *E. coli*. This grouping of bacteria was initially thought to be limited to Gram-positive low GC species, but has since been expanded to include Firmicutes, Fusobacteria, Synergistetes and some Thermotogae and Tenericutes [16,26,31]. In the case of *S. aureus*, this sequence is 9 AAs (amino acids) long (MLKLNLQFF’ASK…), but sequences as long as 15 AAs have been observed (Table 2) [11,32]. Prp is the protease responsible for this essential cleavage [11]. This sequence must be cleaved from L27 before incorporation into the ribosome, or it will prevent tRNA binding through steric hindrance (Figure 1B). This was shown, as *S. aureus* encoding an L27 with an “uncleavable” sequence (MLKLNLQAAASK…) was non-viable. Additionally, *E. coli* expressing *Sa*L27 without Prp was prone to extremely slow growth after protein expression [16].

Most species’ long L27 cleavage sequences follow the following motif: MLxx(D/N)LQ(F/L)F’A(S/H)KK, with xx being a variable domain (Table 2) [16]. However, each species encodes for an L27 with a cleavage sequence that is highly specific to that species’ Prp [12,32]. Purified *Sa*Prp has been seen to cleave a fluorescently labeled *Sa*L27-based peptide (FITC-KLNLQFFASKK-Dnp) with a catalytic efficiency (K_cat_/K_M_) of 74,000 ± 38,000 M^−1^ s^−1^ [32]. This cleavage was not inhibited by a library of common protease inhibitors, including many broad-spectrum covalent cystine protease inhibitors, indicating Prp’s resistance to inhibition. Although the thiophilic compound mersalyl acid was used as a positive inhibition control in this assay, its general toxicity and non-selective nature preclude it from clinical consideration [32,33]. Additionally, the Prp of *C. difficile* was unable to cleave the *Sa*L27-based peptide, further indicating the specificity of Prp to its endogenous substrate [32].

Of note, the proteolytic activity of Prp has not been directly experimentally observed in cellulo for any species other than *S. aureus* and *B. subtilis* [16,34]. There is some evidence of the activity of Prp, however, as no published crystal structures of L27 within a ribosome contains residues N-terminal to the conserved A(S/H)KK motif [35,36,37,38,39,40]. These structures also suggest that addition of residues would reach into the PTC and interfere with protein synthesis. Antibiotics that act at the PTC, such as oxazolidinone, have also been shown to crosslink to the A(S/H)KK motif of L27 in *E. coli* and *S. aureus*, but not to any residues past this point [5,41]. Additionally, the phylogenetic and structural similarities discussed in this paper suggest that Prp activity, at least within the Firmicutes phyla, would be consistent with that seen in *S. aureus*.

## 2. Materials and Methods

### 2.1. Bioinformatics

Alignments of gp46, gp47, L27, Prp, and FlhB peptide sequences were performed using Cluster Omega v1.2.4, available on the UniProt website (uniprot.org, Release 2022_02, The UniProt Consortium [42]) with default settings and zero iterations. Similarity outputs were then used as data for the colored rendering in Table 1, Table 2 and Table 3. FlhB sequences were compared to the L27 cleavage motif [MLxx(D/N)LQ(F/L)F’A(S/H)KK] by hand.

Both the L27 and Prp phylogenic trees were created using the sequence analysis software Geneious 2022.1 (Biomatters, Auckland, New Zealand) available for download at https://www.geneious.com (accessed on 23 June 2022). Accession codes were used to retrieve L27 and Prp sequences on UniProt for representative bacteria of the following phyla: Firmicutes, Fusobacteria, Proteobacteria, Synergistetes, Spirochete, Tenericutes, and Thermotogae. The genomic DNA FASTA sequences were downloaded from the Europena Nucleotide Archive (ENA) database and imported into Geneious. All L27 sequences species of bacteria were aligned using a Geneious global alignment with free end gaps, and a cost matrix of 65% similarity. This alignment was then used to create a neighbor-joining tree model, with no outgroup. The genetic distance model utilized was Tamura-Nei. Branches were selected to be proportionate and ordered.

### 2.2. Structural Analysis

Structural analysis was performed on crystal structures available on the protein data bank (unbound *S. aureus*: 4PEO, bound *S. aureus*: 7KLD, unbound *S. mutans*: 2IDL, unbound *S. pneumoniae*: 2G0J, and unbound *T. maritima*: 1S12). These files were read directly into PyMOL Molecular Graphics System, Version 2.0 Schrödinger, LLC. The waters, ions, and any other molecules used in the crystallization process were removed prior to alignment. In structures containing more than one dimer, the dimer containing the B and D subunits was kept and other atoms removed. All structures were then aligned to the bound *Sa*Prp structure (7KLD) to allow for direct comparison. Measurements were performed with the Wizard measurement tool. Images were taken using the ray setting and modernized rendering. Annotations were made by hand in Microsoft^®^ PowerPoint^®^ Version 2205.

## 3. Results and Discussion

### 3.1. Bioinformatics

The gene for Prp is *ysxB* (previously called DUF464) and is located between the genes for ribosomal proteins L21 (*rplU*) and L27 (*rpmA*). Both *rmpA* and *ysxB* have been classified as essential in *S. aureus* by saturation transposon mutagenesis [43]. The Prp of *S. aureus* (*Sa*Prp) is recognized as the prototypical member of cysteine protease group number 108 (C.108) within clan CR (MEROPS protease database). One of the many benefits of bacterial Prp as an antimicrobial target is that no human analogs exist within the C.108 family or CR clan. The only other enzyme in the CR clan is another cysteine protease that cleaves an N-terminal extension of about 40 amino acids from a capsid protein within Staphylococcal bacteriophage CP-1 [44].

Several phyla of bacteria have been found to contain long L27 through bioinformatic analysis, including Firmicutes, Fusobacteria, Synergistetes, and some Thermotogae and Tenericutes [16,26]. There exists a ribosomal phylogenetic grouping termed Megaphyla III, which includes Firmicutes, Fusobacteria, and Tenericutes. This group is also closely related to Synergistetes, which illustrates the abundance of similarity in the ribosomal characteristics between the phyla containing long L27 [31]. All species encoding long L27 also encode for Prp. The inverse is not true as some bacteria containing Prp do not encode long L27 [16].

**Figure 2:**
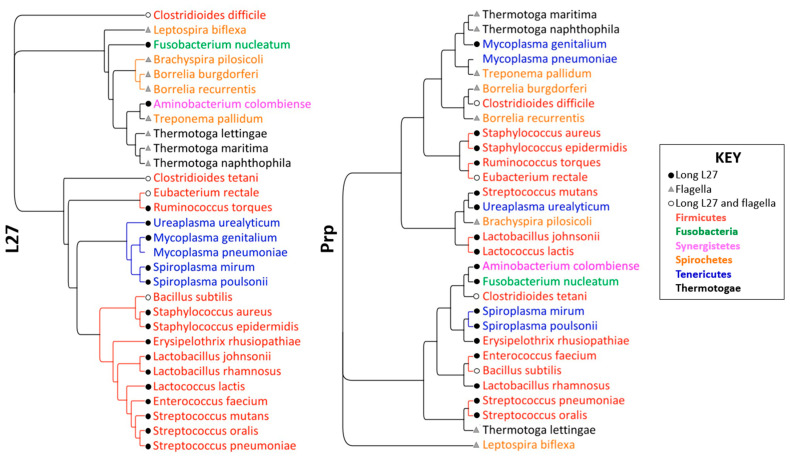
Phylogenic trees of select species of Prp-encoding phyla. (**Left**): L27 sequence comparison. (**Right**): Prp sequence comparison. Image created with Geneious version 2022.1 Biomatters.

Genetic bioinformatic analysis was conducted on selected representative species from the six phyla that encode Prp. The phylogenic tree produced from L27 nucleotide sequence comparison shows good agreement with overall genetic and phylogenic classifications of these species. The L27 tree also shows similar trends in evolutionary relation to the previously published L27 phylogeny by Wall et al. [11]. Of note, this new tree shows multiple bacteria which contain Prp without long L27 (Figure 2). The prevalence of this phenotype indicates an alternative function of the enzyme, such as cleavage of the flagellar protein FlhB (discussed in Section 3.2).

The phylogenic tree comparing nucleotide sequences of Prp revealed a very different organization. Compared to the L27 tree, this phylogeny tells a story of widely varying Prps, even among those phyla that encode for a long L27. In the species that do not contain long L27, such as *T. maritima* and *B. burgdorferi*, Prp does not have the role of cleaving L27, and thus the greater evolutionary differences observed were expected. However, in phyla such as the Firmicutes a conservation in Prp was expected due to the similarity observed in their L27s. This was not the observed phenomena. This may, in part, be due to the L27 cleavage motif differences observed. For example, the Streptococcal species are all highly similar in their L27s (>94% identity), however the *S. mutans* Prp (*Smu*Prp) is significantly different (<50% identity) from that of *S. oralis* and *S. pneumoniae* (97.37% identity between *S. oralis* and *S. pneumoniae*). This is reflected in the eight residues of the L27 cleavage motif proximal to the cleavage point. *S. mutans* has a sequence slightly different (…NLANLQLF’…) to that of *S. oralis* and *S. pneumoniae* (…TLNNLQLF’…). This minor change in L27 results in a significant change required in Prp.

### 3.2. Prps of Bacteria without an L27 N-Terminal Extension

Some species of bacteria encode for Prp without long L27, including all Spirochetes and some Tenericute and Thermotogae [30]. Since these species do not use Prp in ribosomal maturation, targeting these Prps would likely not affect protein synthesis. The endogenous ligand for these Prps has yet to be elucidated; however, Wall et al. proposed FlhB as a potential cleavage candidate in flagellated bacteria [16]. FlhB is a component of the export gate for flagella, as well as the flagellar-derived type III secretion system (T3SS), and is often considered essential for motility [45]. In bacteria that use a T3SS, FlhB is also considered vital for pathogenesis [46]. FlhB is subdivided into two major domains: the N-terminal domain, FlhB_TM_, consists of four transmembrane α-helicies, and the C-terminal domain, FlhB_C,_ which is located in the cytoplasm. FlhB_C_ is known to undergo autocleavage as part of its role in substrate switching, an important part of the T3SS pathogenesis mechanism, and much research on FlhB has focused on this domain. As a result, not much is known about FlhB_TM_ other than its binding position in the FliPQR-FlhB export apparatus. Minamino et al., however, did show that a P28T mutation in the FlhB of *Salmonella enterica* serovar Typhimurium (*S. Typhimurium*) resulted in significantly decreased motility and flagellar protein expression, indicating some role for this region [47].

In *B. subtilis*, a bacterium with both long L27 and flagella, the cleavage sequence for L27 matches almost exactly with a proposed cleavage site in FlhB (MLRLDLQFF… and MKLRVDLQFF…, respectively). Additionally, the FlhB in other bacteria, such as *E. coli*, does not extend past this potential cleavage site (Table 3). This indicates that *Bs*Prp may be doing double duty, cleaving both L27 and FlhB. This pattern of FlhB retaining homology to the long L27 cleavage motif is true for other Firmicutes with both proteins (Table 3), suggesting that both cleavages may be occurring in bacteria with long L27 and the extended FlhB. In bacteria with no long L27, the role of Prp may be exclusive to cleavage of FlhB.

Some bacteria, such as many Spirochetes and Thermotogae, have a Prp-FlhB phenotype (i.e., Prp and FlhB containing, but lacking a long L27). These include the pathogens *B. burgdorferi*, the causative agent of Lyme disease, and *Treponema pallidum*, the causative agent of syphilis. Deep tissue penetration and persistence are common in these pathogens and can lead to lifelong illness [48,49]. FlhB is considered a pathogenesis factor in many pathogens in which it is present, as it is essential for motility and, therefore, infection dissemination throughout the host [50]. If the N-terminal FlhB cleavage by Prp is essential for motility in these pathogens, then a Prp-directed therapy could be used as an adjuvant therapy to prevent deep tissue progression of bacteria.

### 3.3. Mechanism

#### 3.3.1. Proteolytic Mechanism of Prp

**Figure 3:**
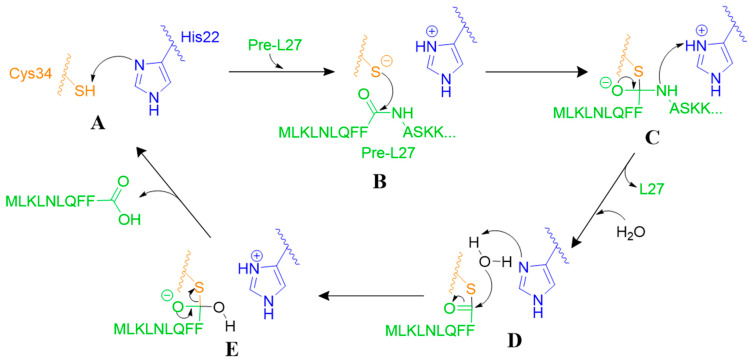
Mechanism of *Sa*Prp proteolysis of long L27 (green) via a canonical cysteine protease catalytic dyad comprising Cys34 (orange) and His22 (blue). (**A**). Initiation via deprotonation of cysteine by the histidine. (**B**). Cystine thiolate acts as a nucleophile to attack the carbonyl carbon of the amide. (**C**). Oxyanion tetrahedral intermediate breaks down. (**D**). Intermediate thioester is attacked by water. (**E**). Cleavage is completed and product released.

Prp is a cysteine protease. Every living organism contains cysteine proteases, as they are crucial to many important cellular functions [51,52,53]. Cysteine proteases have either catalytic triads (Cys-His-Asn) or dyads (Cys-His) that assist in irreversibly cleaving proteins. Cysteine proteases that use a catalytic dyad are initialized by deprotonation of cysteine by the histidine (A). This thiolate acts as a nucleophile to attack the carbonyl carbon of the amide (B) to form an oxyanion tetrahedral intermediate (C), which breaks down to form an intermediate thioester (D), that is subsequently cleaved by water (E) (Figure 3) [54]. Even though cysteine proteases follow this general mechanism, each enzyme contains residues with slightly different sequences, allowing for different substrate specificity. Due to the wide array of functional capabilities of cysteine proteases, they have become a popular drug target in recent years, as they stand as possible treatment options for many diseases [55].

Prps were initially believed to have an Asp-His-Ser catalytic triad, due to the proximity of the residues in the available inactive crystal structures and inactivity of the D31A mutant in vitro studies [11,12]. However, the crystal structure of Prp with a covalently bonded ligand does not support this hypothesis [32]. Instead, this crystal structure shows that Asp31 may play a structural role as it stabilizes the binding site flexible loop using four hydrogen bonds to backbone amides. In addition to these hydrogen bonds, Asp31 also contributes a negative charge that assists in stabilizing the positive amino terminus of the α-helix dipole. In the wild-type protein, the sidechain of this aspartate is located between the catalytic cysteine and the β-loop at His25, shortening the α-helix slightly. Some interactions between Asp31 and Cys34 are possible but unlikely. When Asp31 is lost completely in the D31A mutation, the enzyme’s function is also completely lost, implying that stabilization of this loop is crucial to having a functional active site [11,12].

The prototypical Prp, *Sa*Prp, uses the catalytic dyad of Cys-His to cleave the cleavage motif from long L27 and release mature L27. H22A, C34A, and C34S *Sa*Prp mutants have >95% loss in catalytic activity, implying that these residues are the catalytic dyad used in hydrolysis [11,12]. In initial in vitro experiments, the loss of catalytic activity in the H22A mutant was associated with the loss of the ability to accept a proton from the active site Cys34 (Figure 3A) [12]. However, the recently published covalent complex shows His22 on the opposite side of the substrate than Cys34, implying that accepting a proton from this residue would be very improbable (although not impossible) [32]. A likely alternative mechanism is that His22 acts as a general acid proton donor to the backbone amide on the carboxyl side of the scissile bond that then becomes the leaving group (Figure 3).

In mutating Cys34 to serine, Prp was essentially changed from a cysteine protease to a serine protease. As with most other serine proteases, the high pI of the hydroxyl group within serine requires a proton-accepting group to form the seroxide nucleophilic group that will attack the substrate [56]. Cysteine, however, can form its nucleophilic group through deprotonation by either a proton acceptor or solvent at physiological pH [55]. The option of solvent deprotonation (Figure 3) allows Cys34 to function, even if His22 imidazole is on the opposite side of the scissile bond and, therefore, unable to act as a proton acceptor. This mechanism is strongly supported by the recently published *Sa*Prp crystal structure [32].

#### 3.3.2. Prp as a Ribosomal Protein Chaperone

There is some indication that Prp acts as a chaperone for L27 during ribosomal maturation. The first indication of this was that *S. aureus* encoding “pre-cleaved” L27 (M’ASK…) that is identical to the L27 found in mature ribosomes were found to have significant growth defects [11,12]. Without tight binding to Prp, likely due to the missing cleavage sequence, there are issues with L27 incorporation that result in either misfolding or insufficient mature ribosome assembly [16]. This notion is supported by Prp sharing characteristics with known ribosomal protein chaperones, such as genetic encoding directly upstream of the binding partner and chaperone binding to the ribosomal protein’s N-terminus during translation [57,58,59]. The study of dedicated RP chaperones is underdeveloped, with only 9 of 80 RPs in yeast having specific chaperones and no specific chaperones having been defined in bacteria [58,59].

Overexpression of an *Sa*Prp C34A mutant resulted in growth defects of the bacteria compared to WT Prp. This could be attributed to the 50% decrease seen in mature 70S ribosomes, concurrent with an accumulation of the aberrant pre-50S particles. These particles lacked the late-binding RPs L16, L27, and L25, indicating a ribosome trapped in the late stage of maturation. Interestingly, both L27 and L16 interact directly with tRNA at the PTC, and Western blot analysis of a pre-50S subunit within the C34A Prp showed Prp/L27 complexed with the subunit [12]. This evidence again suggests Prp may play a role in stabilizing the 50S ribosomal subunit or late RP incorporation during maturation.

*Sa*Prp has a high catalytic efficiency (74,000 ± 38,000 M^−1^ s^−1^) relative to other cysteine proteases (approx. 100–25,000 M^−1^ s^−1^) [60,61,62,63]. This is partly due to its low K_m_ (3.4 ± 1.8 μM), which may reflect a tight binding and, therefore, slow k_off_ for the bound cleavage substrate of the *Sa*Prp catalyzed reaction. Also consistent with tight binding is that the cleaved portion of the substrate has been shown to act as a competitive inhibitor to catalysis [12]. This tight binding of L27 after cleavage is consistent with the proposed chaperone function of *Sa*Prp during ribosome maturation.

### 3.4. Structure

**Figure 4:**
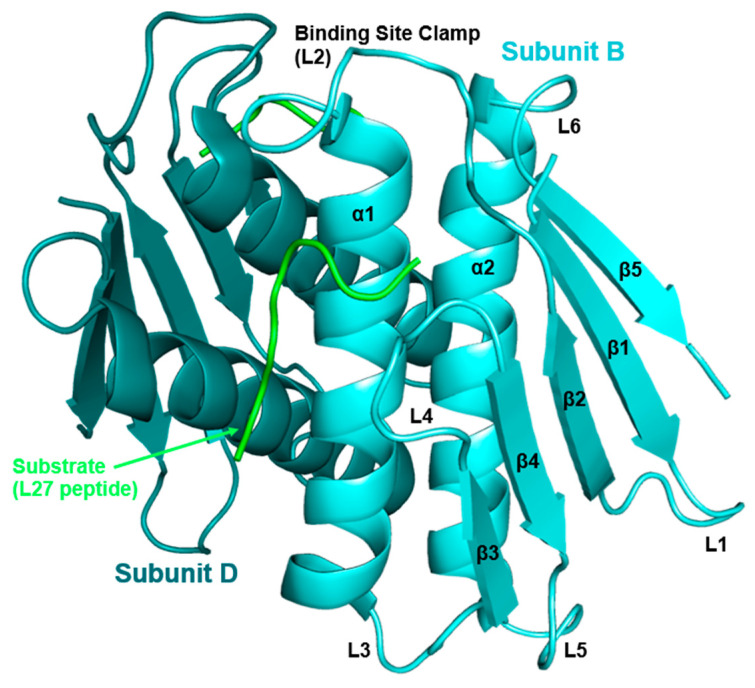
General structure of Prp.

High-resolution three-dimensional molecular structures are often indispensable for drug design [64]. These structures help with the virtual screening and refinement of possible drug candidates. There are currently four bacterial species with published crystal structures of Prp without a ligand in the active site (*S. aureus*: 4PEO, *S. mutans*: 2IDL, *S. pneumoniae*: 2G0J, and *T. maritima*: 1S12). Each of these four structures show the catalytic dyad (His and Cys) at distances or orientations not suitable for catalysis [65,66,67,68]. This variation in conformation is likely due to the flexible loop (Figure 4, L2), which acts as a clamp during catalysis.

After the cleavage function of Prp was determined in *S. aureus* by Wall et al., *Sa*Prp became the founding member of family C.108 [11]. This family is characterized by a novel and unique peptide chain homology of N→C, ββαββαβ (Figure 4). Family C.108 has high fold conservation, approximately 20–30% identity on average, and is conserved among classes: *Bacilli*, *Spirochaetales*, and *Thermotogales* [31]. In 2022, the first crystal structure of Prp bound to substrate was published for *S. aureus* (PDB: 7KLD) and now serves as the basis for comparison of bound to unbound Prp analysis.

#### 3.4.1. Case Study: SaPrp Structural Studies

**Figure 5:**
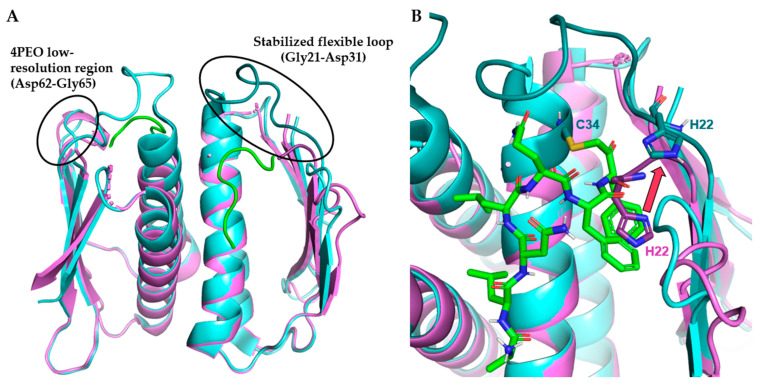
Surface representation of published *Sa*Prp structures. (**A**). Unbound *Sa*Prp (4PEO, violet, 1.73 Å) overlaid with liganded *Sa*Prp (7KLD, cyan, 2.25 Å) bound to covalent to L27-like covalent inhibitor (KLNLQFF-CMK, green). (**B**). *Sa*Prp binding site showing movement of catalytic His22 upon binding (red arrow). Flexible loop and catalytic residues in darker shades. All images produced with PyMOL Molecular Graphics System, Version 2.0 Schrödinger, LLC.

The first high-resolution partial crystal structure of *Sa*Prp (RCSB ID: 4PEO) was published in 2015 [69]. A significant drawback of this structure is the lack of electron density for residues from positions 23–31 (Figure 5A). This missing segment contained Cys34 which, along with His22, makes up the catalytic dyad. This segment was presumed to be flexible and hypothesized to stabilize upon binding the N-terminal pre-L27 sequence, thereby organizing the catalytic site and effecting cleavage. To obtain a fully resolved crystal structure of the binding site to facilitate drug discovery, Hotinger et al. used a peptide substrate containing a chloromethyl ketone (CMK) warhead to covalently link with *Sa*Prp to stabilize the L2 flexible loop (Figure 5) [32]. This resulted in the first bound Prp crystal structure with a substrate bound, and the first *Sa*Prp with a fully resolved L2.

Along with the complete resolution of *Sa*Prp, the new structure provides insights into the bound versus unbound conformation of Prp. Although the His22 of both structures is resolved, the His22 of the unbound structure resides in space that becomes occupied by substrate in the covalently bound structure. The His22 in the bound structure moves out of the way (Figure 5B, red arrow) to allow for the P1–P2 position residues to take its place. Similar restructuring can be seen in the Asp62-Gly65 region, although conclusions cannot be drawn due to the low-resolution nature of the region in the unbound structure.

**Figure 6:**
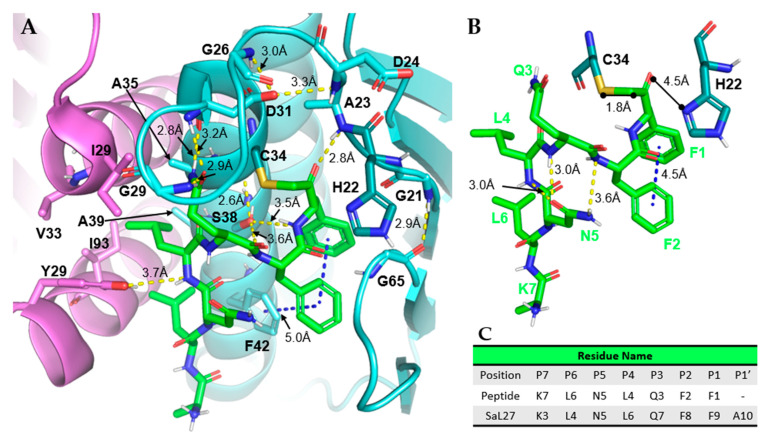
Interactions between acetyl-KLNLQFF-chloromethylketone (termed Ac-KLNLQFF-CH_2_- when covalently bound) and *Sa*Prp. (**A**). Intermolecular forces between peptide (green) and Prp (subunit A is cyan with His22 and Cys34 in teal, subunit D is violet). Noteworthy Prp residues are bolded. (**B**). Interactions between covalently bound peptide (green) and catalytic residues (teal). (**C**). Table with L27 cleavage sequence naming conventions. All images produced with PyMOL Molecular Graphics System, Version 2.0 Schrödinger, LLC. Dashed yellow lines indicate hydrogen bonds, blue dashed lines are π-π interactions.

The 7KLD structure also provided the first glimpse into the binding site interactions made between Prp and L27. Many of the interactions that Prp uses to stabilize its covalently bound substrate are hydrophobic. At the P1–P2 position of the binding site, there is a Phe-Phe which makes an edge-to-face π-π “interaction network” with the Prp’s Phe42 (Figure 6A). It has also been indicated that P1 forms a π-π stacking interaction with His22 of Prp [12]. Without this interaction, His22 would be too mobile to be able to participate in catalysis. Because of this, most known L27 sequences contain phenylalanine in the P1 position (Table 2). Additionally, L4 and L6 occupy desolvated space at the interface of the Prp dimer (Figure 6A). Collectively, these hydrophobic interactions most likely help position the bound peptide correctly for cleavage. Furthermore, at the P1′ position, this structure contains an alanine and illustrates a considerable size limitation at this position of the peptide. The P1′–P2′ substrate positions of known Prp substrates are occupied by small residues such as alanine and serine (Table 1 and Table 2), which further supports the theoretical special limitations.

The P3 position glutamine of the substrate plays two crucial roles in stabilization and binding. The first role stabilizes the flexible loop L2 through hydrogen bonds with Gly29 and Asp31, which places His22 in correct proximity to Cys34 for catalytic activity (Figure 6A). The second role of glutamine is forming an intra-peptide hydrogen bond between the N_ε2_ of glutamine and the carbonyl oxygen of the P4 position leucine (Figure 6B). This stabilizes the peptide in a conformation that can be reacted upon. Glutamine also forms a similar hydrogen bond from its backbone nitrogen to the sidechain amide oxygen of the asparagine at the P5 position (Figure 6B). These hydrogen bonds formed could potentially be the reason for Prp’s high selectivity, as they could help form the confirmation of the complementary peptide to the active site.

Two different residues create the oxyanion hole responsible for stabilizing the transition state of the substrate. Ala23 creates the first half through its backbone amide in the flexible loop (Figure 6A). The imidazole sidechain of His22 completes the other half. This imidazole contains a protonated N_δ1_ that is 4.5 Å away from the thioester carbonyl oxygen of Cys34 that covalently attaches to the bound substrate in this crystal structure (Figure 6B). However, with a true substrate bound, such as L27, the lack of a methylene group bound to Cys34 should orient His22 so that the imidazole sidechain is within hydrogen bond distance to Cys34. The L2 flexible loop is flanked by conserved glycine residues (Gly21 and Gly29) that help preserve the oxyanion hole through their flexibility. This flexibility creates a fluctuating pocket that minimizes the chance for incorrect bond formation, but will stabilize both position and charge once catalysis begins.

The Christie Lab constructed *Sa*Prp mutants to evaluate the importance of active site residues [12]. The G21A, D31A, and S38A mutants were found to be inactive (loss of catalytic activity over 97%), reiterating their importance for binding and/or cleavage [12,70]. Gly21 and Gly29 were hypothesized to be the amino-terminal and carboxyl-terminal hinge points for L2 restructuring, respectively. The loss of Gly21 in the G21A mutation results in a loss of loop flexibility; therefore, this loop cannot be reoriented, blocking the substrate from binding in a cleavable position.

The stabilization of L2 after restructuring is also essential to ligand binding and catalysis. Activity was lost after D31, a residue important to L2 stabilization, was mutated to alanine. Additionally, the inactive G21A mutant Prp may result in a conformation unable to make the intra-protein hydrogen bond between Gly21 and Gly65. This reduces the stability of the catalytically active conformation. Although it is unlikely the loss of this hydrogen bond is the main cause for loss of activity in the G21A mutant, it likely plays a role.

The *Sa*Prp-peptidyl complex crystal structure shows that the serine located on helix α1 (Ser38) forms several crucial interactions between the substrate and surrounding residues (Figure 6A). The first of these interactions is a 2.6 Å hydrogen bond with the carbonyl oxygen within Cys34, likely holding it into catalytic conformation. Ser38 also forms two hydrogen bonds of 3.5 Å and 3.6 Å with the backbone amides of the residues at substrate positions P1–P2. Although these interactions are just outside the range of a typical hydrogen bond, they are likely shorter when interacting with the endogenous ligand rather than chloromethylketone, because this inhibitor contains a methyl group the endogenous ligand does not. The function of Ser38 is essential; the interaction with Cys34 stabilizes the orientation of the catalytic amino acid, and the hydrogen bonds with P1–P2 peptide linkage allow P1 to interact with Cys34 to be cleaved.

#### 3.4.2. Firmicute Prp Homology

**Figure 7:**
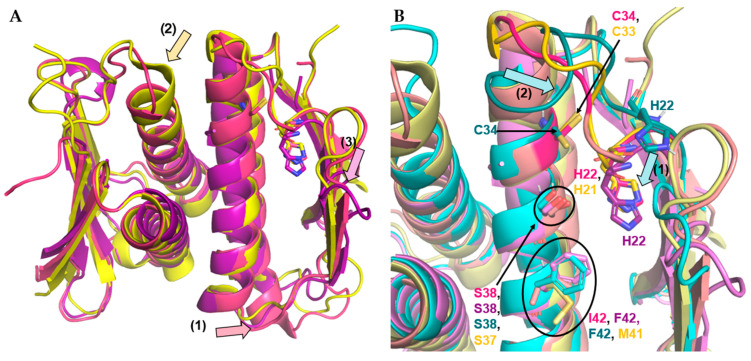
Firmicute Prp structures. (**A**). Unbound Firmicute Prp crystals overlaid. *Sa*Prp (4PEO) in violet, *Smu*Prp (2IDL) in pink, and *Sp*Prp (2G0I) in yellow. Significant changes indicated by arrows and catalytic residues shown as stick representations. (**B**). Binding site of all known Firmicute Prp structures, unbound *Sa*Prp, *Smu*Prp, and *Sp*Prp and bound *Sa*Prp (7KLD, cyan). Flexible loop and catalytic residues in darker corresponding colors. Movement attributed to binding indicated by blue arrows (1 and 2). All images produced with PyMOL Molecular Graphics System, Version 2.0 Schrödinger, LLC.

The similarity of Firmicute Prps in structure is evident when comparing the position of the catalytic residues and other major binding site residues of the available structures. In the unbound structures, the catalytic histidine and cysteine in each of the structures line up and have highly similar conformations (Figure 7A). When overlaid with the unbound *Sa*Prp partial crystal structure, *Smu*Prp and *Sp*Prp (Figure 7A) showed similar sequences and confirmations with three minor exceptions: a full turn extension on the N-terminal end of α2 in *Smu*Prp (Figure 7A, 1), elongation of the C-terminal end of α1 in *Sp*Prp (Figure 7A, 2), and the different L2 conformation taken by *Sa*Prp (Figure 7A, 3). These differences do not significantly affect the substrate binding site but may cumulatively result in changes in cleavage sequence preferences. As an example, the extended α1 in S*p*Prp may allow for more space in the P1′–P2′ area to allow for the slightly larger histidine residue in the P2′ position area (MTLNNLQLF’AHKK…) in comparison to the serine in *Sa*L27 (MLRLDLQFF’ASKK…).

This comparison of structures within a phylum also allows for further evaluation of the effect of L27-Prp binding. This is because the *Smu*Prp and *Sp*Prp flexible loops can provide insights into the placement of the *Sa*Prp flexible loop when in an unbound conformation. The mobile segment of L2 in *Smu*Prp rests within the active site, not only preventing substrate binding but also displacing the catalytic His22 residue, causing this confirmation of *Smu*Prp to not be functional (Figure 7B, 1). The His22 can be shifted into the active site through the movement of the mobile segment along with the substrate binding of positions P1–P3. This binding of the P1–P3 positions could be one of the contributing factors to the high specificity of Prp towards the specific pre-L27 within the bacteria (or Prp inhibitors). Additionally, the terminus of α1 is unwound for nearly one full turn on the distal end in the unbound *Smu*Prp (Figure 7B, 2). This region coils much tighter in the bound *Sa*Prp to allow for the mobility of the Gly21-Gly26 segment to move out of the active site which in turn supports substrate binding.

Another example of this is the stabilizing Gly-Gly hydrogen bond between the C-terminus of L4 and N-terminus of L2. In unbound *Smu*Prp, the carboxyl oxygen of Gly66 interacts with the backbone nitrogen of Gly21 at a distance of 3.1 Å. A very similar bond is found in the bound Prp structure (2.9 Å). The unbound *Sa*Prp structure, however, has 7.3 Å between these atoms, as Gly65 is facing the opposite direction. Although this may be due to poor resolution in this region, it may also reflect some of the restructuring undergone by *Sa*Prp upon interaction with L27.

Along with the catalytic residues, other conserved binding site residues, such as Ser38 and Phe42 on the α1 helix, can be compared in the context of binding (Figure 7B). Of note, the Phe42 of *Sa*Prp was previously thought to be conserved, as it makes important interactions with *Sa*L27 during binding. This position is, however, seen to be variable, as the *Smu*Prp has isoleucine at residue 42 and the *Sp*Prp has methionine at residue 41. Changes at these binding site residues may be the cause of some of the selectivity that has previously been observed.

#### 3.4.3. Non-L27 Cleaving Prps

**Figure 8:**
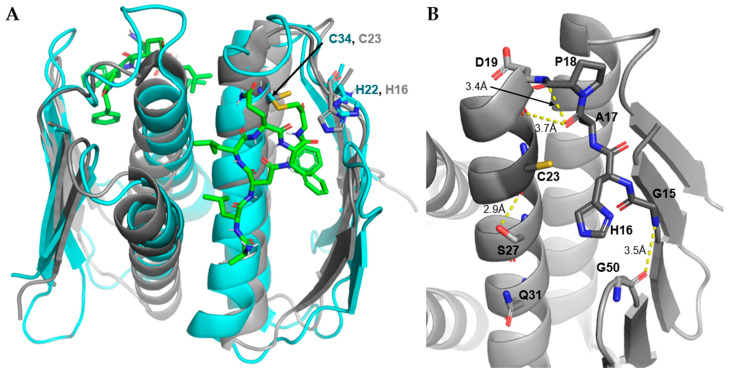
Non-L27 cleaving Prp structure. (**A**). Cartoon representation of *Sa*Prp (7KLD, cyan) bound to substrate (green) aligned with *Tm*Prp (1S12, gray). Proposed catalytic residues shown as sticks. (**B**). Binding site of *Tm*Prp with stabilizing hydrogen bonds (yellow dashed lines). All images produced with PyMOL Molecular Graphics System, Version 2.0 Schrödinger, LLC.

The Prp in bacteria that do not have long L27 tend to have shorter flexible loops, potentially allowing for larger and less specific substrates or due to lack of use of the protease [11,16]. These Prps also tend toward more neutral isoelectric points (PIs) as opposed to the acidic nature of Firmicute Prps (e.g., *Sa*Prp = 4.45) [11,16,32]. There currently exists only one Prp crystal structure of a non-L27 cleaving Prp, that of *T. maritima* (1S12). The catalytic residues of this structure align with the corresponding residues within the bound *Sa*Prp crystal structure (Figure 8). This is likely due to the very short nature of its L2, measuring half the residues (Gly15-Asp19) in comparison to that of *Sa*Prp (Gly21-Asp31). Of note, the Pro18 of *Tm*Prp appears to allow for a sharp turn in the loop, allowing for His22 to rest in an active conformation. This is diametrically opposed to the resting/unbound pose of other Prps, as they relax into a conformation unfit for catalysis (Figure 7).

Many of the stabilizing interactions seen in the Firmicute Prp structures can be seen in the *Tm*Prp structure (Figure 8B). This includes the hydrogen bond between Gly15 and Gly50 that stabilizes the distal end of L2. The conserved α1-helix serine is in a slightly different orientation but is still within range (2.9 Å) to make a hydrogen bond with the backbone of the catalytic cysteine. The proximal end of L2 is stabilized by Asp19 through a hydrogen bond between the backbones Asp19 and Pro18 (3.4 Å). Interestingly, there is a distance of 3.7 Å between the carboxyl oxygens of Asp19 and Pro18. This distance would provide sufficient space for a water-bridged hydrogen bond. This bridged interaction may allow for the stabilization of the water molecule used in catalysis, as it would be held in proximity to the cleavage site of a bound substrate.

## 4. Conclusions

Prp is a recently characterized enzyme used by Firmicutes, Fusobacteria, Synergistetes, Spirochetes, Tenericutes, and Thermotogae. It has been shown to be essential in the pathogen *S. aureus,* making it a potential new antibiotic target. It is the prototype member of C. 108, which has no human homologs. Since only a subset of bacteria encode Prp, it is an attractive target for selective drug design. This narrow-spectrum design would slow resistance development due to lower selective pressures across all bacteria.

Our bioinformatic analysis reveals and emphasizes the coevolutionary relationship between Prps and their cleavable L27 sequences. Notwithstanding this critical relationship, FlhB was also found to have potential as a substrate, with the N-terminus of FlhB and L27 in *B. subtilis* having the same cleavage motif. The recently published crystal structure with a covalently linked substrate has allowed for structural insights explaining observed reduced catalytic activity in active site mutants. This has facilitated the structural comparison with unbound Prps of multiple species, giving insights into their homology and differences. These insights reveal the selective nature of Prp-L27 interactions and may guide future pathogen-specific drug design.

**Accession Codes:**

Proteins used in alignments: gp46, A4ZFB2; gp47, A4ZBF3; **L27:** *Ac*L27, D5EEJ4; *Bb*L27, O51721; *Bp*L27, D8IEK6; *Br*L27, B5RQB7; *Bs*L27, P05657; *Cd*L27, Q18B22; *Ct*L27, Q892N6; *Ec*L27, P0A7L8; *Ef*L27, A0A132ZFM0; *Ere*L27, C4Z9Z8; *Erh*L27, E7FUU3; *Fn*L27, Q8REI3; *Lb*L27, B0SRT6; *Lj*L27, Q74IL5; *Ll*L27, Q9CGL5; *Lr*L27, K8QHR9; *Mg*L27, P47476; *Mp*L27, AAB96157.1; *Rt*L27, A5KLI2; *Sa*L27, Q2FXT0; *Se*L27, Q8CS89; *Smi*L27, W6AKZ4; *Smu*L27, Q8DUQ4; *So*L27, F2QE23; *Spn*L27, P66136; *Spo*L27, A0A2P6FBS6; *Tl*L27, A8F8P7; *Tm*L27, Q9X1G7; *Tn*L27, ADA67440.1; *Tp*L27, B2S3Y2; *Uu*L27, B5ZB18; **Prp:** *Ac*Prp, D5EEJ5; *Bb*Prp, A0A0H3C0W4; *Bp*Prp, D8IEK5; *Br*Prp, B5RQB6; *Bs*Prp, P26942; *Cd*Prp, A0A031WH70; *Ct*Prp, A0A4Q0V4F9; *Ef*Prp, Q3XX33; *Ere*Prp, R6TK63; *Erh*Prp, E7FUU4; *Fn*Prp, A0A2G7H7J2; *Lb*Prp, B0SRT5; *Lj*Prp, F4AH23; *Ll*Prp, A0A0H1RUX4; *Lr*Prp, A0A171J8E4; *Mg*Prp, P47475; *Mp*Prp, P75459; *Rt*Prp, D4M5X3; *Sa*Prp, Q2FXS9; *Se*Prp, A0A0N0LXG5; *Smi*Prp, W0GKZ6; *Smu*Prp, Q8DUQ5; *So*Prp, A0A139PD13; *Spn*Prp, C1C7A0; *Spo*Prp, A0A2P6FBV9; *Tl*Prp, A8F8P8; *Tm*Prp, Q9X1G8; *Tn*Prp, D2C3Y5; *Tp*Prp, A0A0H3BLA2; *Uu*Prp, B5ZB19; **FlhB:** *Bb*FlhB, Q44760; *Bp*FlhB, D8IA38; *Br*FlhB, B5RR92; *Bs*FlhB, P35538; *Cd*FlhB, A0A0H3MZI1; *Ct*FlhB, A0A4Q0VFP5; *Ec*FlhB, P76299; *Er*FlhB, D6E4Y1; *Lb*FlhB, B0SLZ8; *S*TFlhB, P40727; *Tl*FlhB, A8F4V6; *Tm*FlhB, Q9X011; *Tn*FlhB, D2C590; *Tp*FlhB, O83710

Genes used in phylogenic tree creation: **L27:** *Ac*L27, ADE56976; *Bb*L27, AAC67128; *Bp*L27, ADK31579; *Br*L27, ACH95001; *Bs*L27, CAA26492; *Cd*L27, CAJ68016; *Ct*L27, AAO36559; *Ec*L27, BAA02526; *Ef*L27, AQY30577; *Ere*L27, ACR75439; *Erh*L27, EFY09659; *Fn*L27, AAL95315; *Lb*L27, ABZ97881; *Lj*L27, AAS09323; *Ll*L27, AAK05179; *Lr*L27, EKS52799; *Mg*L27, AAC71455; *Mp*L27, AAB96157; *Rt*L27, EDK24820; *Sa*L27, ABD30824; *Se*L27, AAO04927; *Smi*L27, AHI57832; *Smu*L27, AAN58565; *So*L27, CBZ00892; *Spn*L27, AAK99818; *Spo*L27, PQM30923; *Tl*L27, ABV34531; *Tm*L27, AAD36524; *Tn*L27, ADA67440; *Tp*L27, ACD71161; *Uu*L27, ACI59937 **Prp**: *Ac*Prp, ADE56977; *Bb*Prp, ACK74753; *Bp*Prp, ADK31578; *Br*Prp, ACH95000; *Bs*Prp, CAA42109; *Cd*Prp, CDS85844; *Ct*Prp, RXI40396; *Ef*Prp, AFK58390; *Ere*Prp, CDC73813; *Erh*Prp, EFY09660; *Fn*Prp, ATV05802; *Lb*Prp, ABZ97880; *Lj*Prp, AEB93126; *Ll*Prp, ARD98746; *Lr*Prp, NZA04214; *Mg*Prp, AAC71454; *Mp*Prp, AAB96158; *Rt*Prp, CBL26635; *Sa*Prp, ABD30825; *Se*Prp, MBF9303503; *Smi*Prp, AHI57831; *Smu*Prp, AAN58564; *So*Prp, KXT86311; *Spn*Prp, ACO17375; *Spo*Prp, PQM30922; *Tl*Prp, ABV34532; *Tm*Prp, AAD36525; *Tn*Prp, ADA67439; *Tp*Prp, ACD71162; *Uu*Prp, ACI60250

## Figures and Tables

**Table 1:** The amino acid sequence present in Staphylococcal phage 80α aligned with the sequence of L27 in *S. aureus*.

Name	Sequence
80α Scaffold (gp46)	ME-ENKLKFNLQFF’ADQS…
80α Capsid (gp47)	MEQTQKLKLNLQHF’ASNN…
*S. aureus* L27	M-----LKLNLQFF’ASKK…

High conservation in gold, moderate conservation in light yellow, Alignments performed with Cluster Omega.

**Table 2:** L27 N-terminal extension cleavage sequence comparison.

Phylum	Species	L27 N-Terminus
Firmicutes	*Bacillus subtilis*	MLRL------DLQFF’ASKK…
	*Clostridioides difficile* ^†^	MLNM------NLQLL’ASKK…
	*Clostridium tetani* ^†^	MLLM------NLQLF’ATKK…
	*Enterococcus faecium*	MLLSM-----NLQLF’AHKK…
	*Erysipelothrix rhusiopathiae*	M-KF----VLDIQLF’ASKK…
	*Eubacterium rectale*	MLNM------NLQFF’AHKK…
	*Lactobacillus johnsonii*	MM---INNLEALKLF’AHHK…
	*Lactobacillus rhamnosus*	MLKM------NLQFF’SHHK…
	*Lactococcus lactis*	MLEL------NLQLF’AHKK…
	*Staphylococcus aureus* ^†^	MLKL------NLQFF’ASKK…
	*Staphylococcus epidermidis*	MLKL------NLQFF’ASKK…
	*Streptococcus mutans* ^†^	MLKM---NLANLQLF’AHKK…
	*Streptococcus oralis*	MLKM---TLNNLQLF’AHKK…
	*Streptococcus pneumoniae* ^†^	M------TLNNLQLF’AHKK…
	*Ruminococcus torques*	MMKM------NLQFF’AHKK…
Fusobacteria	*Fusobacterium nucleatum*	M-----QFLFNIQLF’AHKK…
Synergistetes	*Aminobacterium colombiense*	M----RINFFDLQFF’AHKK…
Spirochete	*Brachyspira pilosicoli* ^†^	M--------------’AHKK…
	*Borrelia recurrentis* ^†^	M--------------’ATSK…
	*Borrelia burgdorferi* ^†^	M--------------’ATSK…
	*Leptospira biflexa*	M--------------’ATKK…
	*Treponema pallidum* ^†^	M--------------’AR-K…
Tenericutes	*Mycoplasma genitalium* ^†^	MSKNSYCYQINLQFF’ASKK…
	*Mycoplasma pneumoniae* ^†^	M--------------’ASKK…
	*Spiroplasma mirum* ^†^	MMKFL----LGLQLF’ASKK…
	*Spiroplasma poulsonii*	MMKFL----LGLQLF’ASKK…
	*Ureaplasma urealyticum*	MNKL--YWLTDLQLF’ASKK…
Thermotogae	*Thermotoga lettingae*	M-------RIDIQLF’AHRK…
	*Thermotoga maritima*	M--------------’AHKK…
	*Thermotoga naphthophila*	M--------------’AHKK…
Proteobacteria	* Escherichia coli	M--------------’AHKK…

† Obligate pathogen, * does not have Prp, high conservation in green, moderate conservation in light green. Alignments performed with Cluster Omega.

**Table 3:** FlhB and L27 N-terminal extension cleavage sequence comparison.

Phylum	Species	Protein	FlhB
Firmicutes	*B. subtilis*	L27	M-LRLDLQFF’A-SKK…
		FlhB	MKLRVDLQFF’AG-----EKTEKATPKKRKDTRK-KGQVAKS…
	*C. difficile* ^†^	L27	M---LNMNLQLL’A-SKK…
		FlhB	M…FALAPMFF’MGSTD---KTEEATPKKKGEQRK-KGNIAKS…
	*C. tetani* ^†^	L27	M-LLMNLQLF’A-TKK…
		FlhB	M…VPPILFIF’A-SED---KTEEATPHKLQEARK-KGQVAKS…
	*E. rectale*	L27	M-LNMNLQFF’A-HKK…
		FlhB	M…LCYNLQWF’AQDGEGGEKTEPATEKKLKDARE-EGKVAKS…
Spirochete	*B. pilosicoli* ^†^	FlhB	M…RGFALTLF’ASAEDEG-RTELPTERKKRRAREEEGRVVNS…
	*B. recurrentis* ^†^	FlhB	M…WYIPLNFF’ASE-DEG-RTEVPTEQRKQKARR-EGQVLKS…
	*B. burgdorferi* ^†^	FlhB	M…WYIPLDFF’SAD-DEG-RTELPTDQKKQKARE-EGRVLKS…
	*L. biflexa*	FlhB	M…YEIQLQLF’AAA-DEG-RTEPPSERRRREEKE-KGNVPKS…
	*T. pallidum* ^†^	FlhB	M…FIIDLQWF’AAE-DEG-RSEDPTETKLRKARE-EGRVPKS…
Thermotogae	*T. lettingae*	FlhB	M…KKLFKQLF’ADP--E--KTEKPTPRRRRKARE-EGQVATS…
	*T. maritima*	FlhB	M…IE--LLLF’AEA--E--RTERATPRKRRRVRE-EGRAPVS…
	*T. naphthophila*	FlhB	M…IE--LLLF’AEA--E--RTERATPRKRRRVRE-EGRAPVS…
Proteobacteria	* E. coli	FlhB	M---------’SDESDD--KTEAPTPHRLEKARE-EGQIPRS…
	* *S. Typhimurium* ^†^	FlhB	M---------’AEESDDD-KTEAPTPHRLEKARE-EGQI**P**RS…

† Obligate pathogen, * does not have Prp, sequences following the L27 cleavage motif in green, high FlhB conservation in dark blue, moderate FlhB conservation in blue, low FlhB conservation in light blue. Bolded red residues indicate positions known to be important for motility. Alignments performed with Cluster Omega.
